# Comparison of Caudal Block vs. Penile Block vs. Intravenous Fentanyl Only in Children Undergoing Penile Surgery: A Prospective, Randomized, Double Blind Study

**DOI:** 10.3389/fped.2021.654015

**Published:** 2021-03-26

**Authors:** Margaret Ekstein, Avi A. Weinbroum, Jacob Ben-Chaim, Eyal Amar, Reut Schvartz, Yifat Klein, Yuval Bar-Yosef

**Affiliations:** ^1^Department of Anesthesiology & Critical Care & Pain, Tel Aviv University, Tel Aviv, Israel; ^2^Department of Pediatric Urology, Tel Aviv University, Tel Aviv, Israel; ^3^Department of Orthopedics, Tel-Aviv Medical Center Affiliated With Sackler Medical School, Tel Aviv University, Tel Aviv, Israel

**Keywords:** anesthesia, penis (MeSH), pediatrics - children, surgery, pain

## Abstract

**Objectives:** Penile surgery is commonly performed in pediatric surgical centers. There is no consensus regarding which analgesic method is most effective in controlling pain in these children.

**Methods:** Consecutive children between 4 months and 16 years of age who underwent elective penile surgery were recruited. After inhaled induction of anesthesia, children were randomized to one of three methods of intraoperative analgesia: caudal block, IV fentanyl titrated to surgical response and spontaneous respiration, or dorsal penile nerve block (DPNB). All patients were given inhaled agents; fentanyl was added if either block was insufficient. Demographic data, analgesic use and pain scores were recorded by a blinded investigator in the PACU and ward. Pain scores, analgesic requirement, and recovery parameters of returning to normal activity level, eating, and voiding post-operatively for up to 4 days, were compared.

**Results:** 116 children were recruited. Pain scores in the post anesthesia care unit were significantly lower in the DPNB and caudal block groups compared to the fentanyl group for the first 30 postoperative min. Pain scores and analgesic use were subsequently similar among the three groups for the rest of the study period. There was no statistical difference in time to eat, return to normal activity or in parental satisfaction scores among the groups. There was a trend toward earliest time to void in the DPNB group.

**Conclusions:** Regional blocks most effectively controlled pain for 30 min after surgery. The choice of intra-operative analgesia protocol had no effect on later pain and recovery parameters.

## Introduction

Penile surgery, including circumcision, revision of circumcision and repair of penile torsion, chordee or hypospadias, is commonly performed in pediatric surgical centers. Analgesic methods include caudal, penile, pudendal and ring blocks, opioid and non-opioid systemic analgesics, as well as application of topical agents. Despite the importance of pain control in these patients, there is no consensus regarding which analgesic method is most effective and safe in controlling perioperative pain in these children (1, 2). Regional anesthesia provides effective pain relief with minimal effects on respiratory drive or hemodynamic stability; however, it is associated with occasional but significant complications, especially in infants, and has not been shown to be consistently superior to systemic analgesia in prospective studies (3). Literature asserts that regional anesthesia in infants, while considered safe, should be used with caution (4). Recent retrospective studies have compared different forms of analgesia in children undergoing penile surgery (5); however, prospective studies are rare and few evaluate outcome parameters longer than the immediate post-operative period. Hence, there is a need to clarify which anesthesia option is most effective for post-operative pain relief while providing lasting enhanced recovery after penile surgery in children. The study hypothesis was that blocks, specifically caudal and dorsal penile nerve block (DPNB), are effective in reducing intra-operative and post-operative pain.

We studied children between 4 months and 16 years of age who underwent elective penile surgery under general inhaled anesthesia using three different methods of analgesia: caudal block, intravenous fentanyl or dorsal penile nerve block (DPNB). The primary aim of this prospective, randomized double blind study was to compare pain scores and need of supplemental analgesics in the immediate post-operative period. The secondary aim was to compare recovery parameters up to 4 days post-operatively, as well as overall parental satisfaction scores.

## Materials and Methods

**Figure 1:**
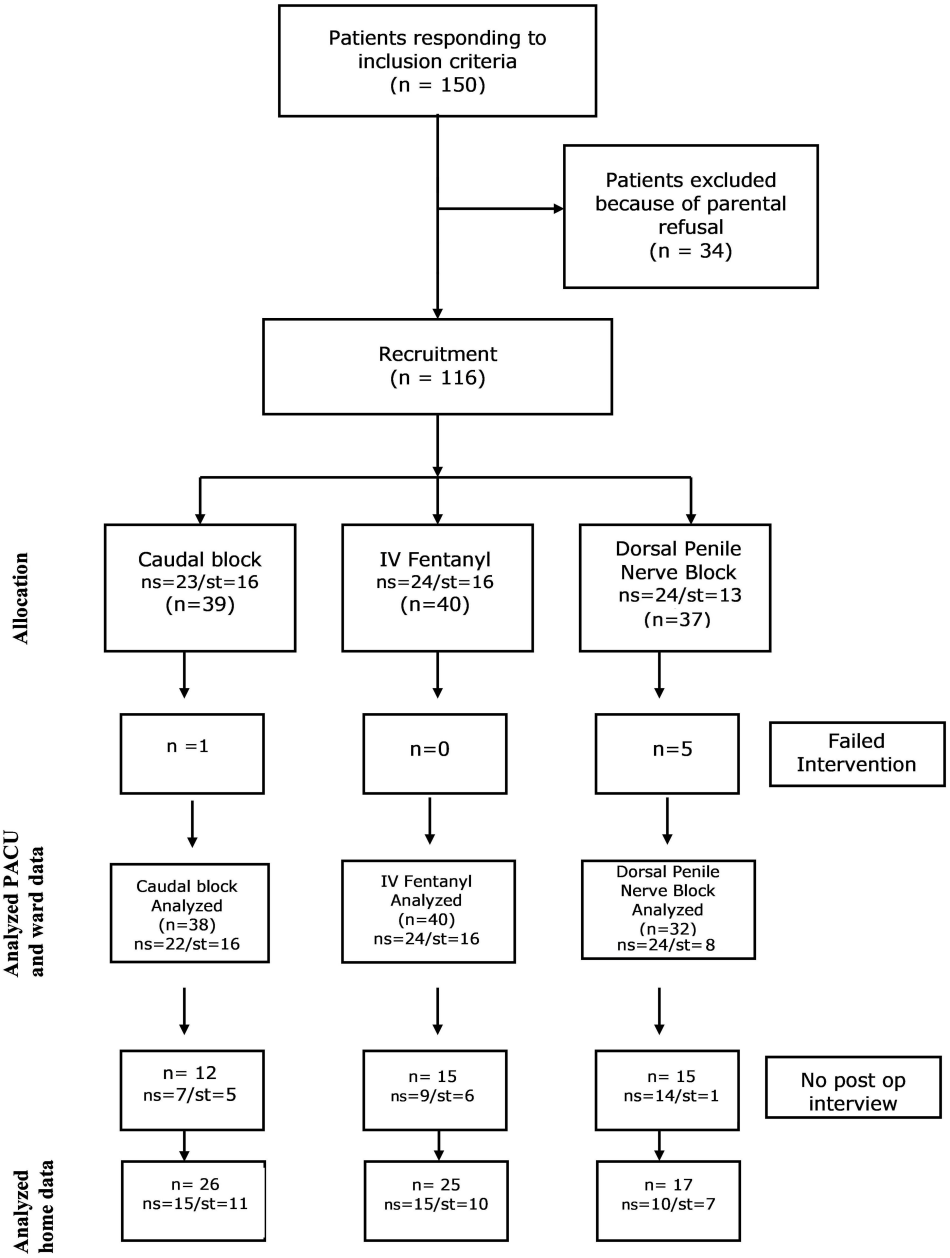
Flow diagram of the study cohort. ns, no penile stent placed; st, penile stent placed; PACU, post anesthesia care unit.

This study was designed as a randomized, double blind, prospective clinical trial, with three arms, and was conducted in accordance with the CONSORT guidelines (Figure 1). After obtaining approval from the local IRB as well as the State Ministry of Health, we recruited consecutive children between the ages of 4 months (at least 60 weeks post conceptual age) and 16 years, undergoing various penile surgeries. Informed consent was obtained in each case from parents or guardians. Exclusion criteria were ASA status >II, a history of bleeding disorder, spinal cord disease, obstructive sleep apnea, developmental delay, behavior disorder, allergy to any of the protocol's medications, or chronic cardio/pulmonary disease which resulted in chronic hypoxemia. Patients were also excluded if their penile surgery was part of a combined procedure with another operation (e.g., hernioraphy, hydrocelectomy, etc.) or if surgery was expected to include intervention at another site (e.g., buccal mucosa for hypospadias repair). All children received general anesthesia. Following anesthesia induction, the patients were randomized to one of the three groups as described below.

### Protocol for General Anesthesia

Children were kept fasting according to institutional guidelines. In the holding area they were premedicated with oral midazolam 0.5 mg/kg, not to exceed 15 mg.

Anesthesia was induced in all children with O_2_, N_2_O and sevoflurane. Routine monitors (pulse oximeter, three lead ECG, non-invasive blood pressure cuff and gas analyzer) were used. A laryngeal mask airway (LMA) was then placed while the patient was breathing spontaneously and was sufficiently anesthetized; an intravenous (IV) line was then placed. All patients were given 10 cc/kg IV lactated ringers solution (LR) over the first h of surgery and maintenance fluid for the subsequent hours of surgery. If blood losses exceeded an estimate of 2 cc/kg, they were replaced with LR at a ratio of 3:1.

Anesthesia was maintained with 40% O_2_ in N_2_O and isoflurane at inspired fractions of 0.6–1% under spontaneous ventilation with pressure support 7–10 cmH_2_O. If apnea occurred, or end tidal pCO_2_ rose above 65 mmHg, or if O_2_ saturation dropped below 94%, controlled ventilation was started via the LMA. Intubation was performed if ventilation or oxygenation did not improve the latter. No muscle relaxants were given unless needed as an emergency treatment of laryngospasm. At the end of surgery, inhaled agents were discontinued, the LMA was removed, and the patient was allowed to awaken breathing 100% O_2_ via a face mask. The patients were brought to the PACU while breathing room air.

### Randomization

After induction, patients were randomized via slips of paper pulled from an envelope by the circulating OR nurse to one of three methods of analgesia: (1) caudal block using 0.6 cc/kg of 0.25% bupivacaine (group C); (2) IV fentanyl of 1 mcg/kg increments titrated to lack of movement or hemodynamic response to surgical stimuli, and targeted to a spontaneous respiratory rate of 12–24/min according to patient's age (group F); (3) dorsal penile nerve block injection of 0.25 cc/kg (not to exceed 8 cc) of 0.5% bupivacaine, using the landmark technique (group P). A block was considered a “failed block” if the patient responded (by movement or changes of hemodynamic parameters) to incision, despite 15 min having elapsed from time of block placement to skin incision (during which the patient was repositioned on the surgical table, prepped, and draped). These children were diverted to receive general anesthesia with IV fentanyl as needed and their data were eliminated from that time onward; only their demographic data were included. The same anesthesiologist (ME) performed all intraoperative anesthetics but was not involved in the later data collection or analyses.

### Post Anesthesia Care Unit (PACU) Protocol

A dedicated PACU nurse, blinded to the method of each child's analgesia, followed patients' recovery in the PACU. She/he recorded oxygen saturation on room air, heart rate, respiratory rate every 15 min, as well as any untoward events (including nausea/vomiting) and medications given. In addition, the blinded nurse assessed pain using two scores: (1) Objective scale: FLACC score (scale of 0–10 based on the child's Face, Arm movement (substituted for Leg movement), Activity, Crying and Consolability, each graded between 0 and 2 points, and (2) the subjective numerical rating scale (NRS) as assessed by the parents on a scale of 0 = no pain to 10 = the worst pain possible. The nurse also recorded the sedation status of the child on a four point sedation scale of 0–3, in which 0 = completely awake; 1 = responds to voice; 2 = responds to touch, 3 = does not respond to touch.

Pain and sedation scores were recorded upon arrival to the PACU (time 0) and at 10, 20, 30 and 60 min later. If pain score by either scale was >3/10 and/or the parent requested medication for the child, 0.05 mg/kg of IV morphine was administered. Additional doses were available every 10 min, up to a total of 0.2 mg/kg. Ondansetron 0.15 mg/kg IV was available if there were signs of nausea or vomiting. The child was allowed to drink if fully awake, starting from 10 min after arrival to the PACU. Following existing directives, all children stayed in the PACU for a minimum of 60 min; they could stay longer if still sleepy or in pain.

### Ward Protocol

After discharge from the PACU, patients were admitted to the pediatric surgery ward. Pain and sedation scores were recorded upon arrival (time 0), and 2, 4 and 6 h after arrival by the ward nurse, the parent being consulted every time. If pain score by either scale was >3/10, 10 mg/kg ibuprofen syrup was given to the child; if this was insufficient to control pain after 30 min, 0.2 mg/kg oxycodone syrup was added. Those who refused or were unable to drink 4 h after arriving to the ward were re-attached to IV fluids of 5% Dextrose in 0.45% normal saline for maintenance, until they started to drink. The time to first analgesic, time to drink, eat and stand/walk (age dependent), as well as time to first void, were recorded by a blinded research assistant. All medications given, and any untoward effects, were recorded by the ward nurse.

Those who did not have a urethral stent inserted during surgery, were discharged home the same day of surgery, provided they drank, were able to walk (age dependent), had adequate pain control on oral medications, and had voided spontaneously. Time to spontaneous voiding was recorded. Children who had a urethral stent inserted intraoperatively were discharged the next day, when meeting the same criteria except for voiding requirement.

### Telephone Interview

On post-operative day (POD) 4, the blinded research assistant (YK) called the parent (or guardian) for a short interview. Number of doses of analgesics, and the times of their administration, were recorded. Recovery parameters, consisting of when the child returned to his normal eating pattern and to his normal level of activity, and an overall parental satisfaction score (0–10) were also recorded.

### Data Collection

Data was collected by a blinded research assistant (YK) who followed each child once consent was obtained, throughout the perioperative period, including the telephone interview on POD 4. Data included demographics: age, weight, ASA status, surgical procedure, surgical time and total operating room time, as well as PACU and ward parameters as described. Patients were dropped out of the study if parents requested it at any time, if the procedure was canceled, or changed to one of those listed in the exclusion criteria after randomization was performed.

### Statistical Analysis

Statistical analysis of the data was carried out for categorical variables with the χ2 and Fisher exact test where appropriate, for comparison of mean of scale variables ANOVA was used and Tukey test was used for *post hoc* analysis. Kruskal-Wallis nonparametric test was used when appropriate. A significance level of .05 was used. All analyses were carried out using the IBM® SPSS® 21 for Windows.

## Results

Out of 150 children responding to inclusion criteria, due to last minute parental refusal, 116 children between the ages of 4 months and 15 years were recruited. 39 were blindly randomized to the caudal block group (group C), 40 to the fentanyl group (group F), 37 to the dorsal penile nerve block group (group P). Of the two block groups, six had failed blocks and were given fentanyl in the operating room; their pain and recovery data were excluded from analysis. Interviews on POD 4 were obtained in 68 patients (see consort statement, Figure 1).

There was no difference in the mean age, weight, ASA status, surgical or total OR time among the groups (Table 1). All patients were managed intra-operatively with LMA's; no child required intraoperative intubation. No child required PACU stay longer than the expected time, nor was anyone brought back to the OR within 4 days of surgery for revision of surgery.

**Table 1:** Demographic and surgical data.

**Variables**	**Caudal block** **(*n* = 39)**	**Dorsal penile nerve block** **(*n* = 37)**	**Fentanyl** **(*n* = 40)**
Age (months)	34 ± 36	44 ± 46	27 ± 29
Range (months)	(4–132)	(6–166)	(5–‘139)
Weight (kg)	15 ± 9	17 ± 11	13 ± 6
Range (kg)	(7–46)	(8.6–43)	(6.9–33)
ASA status: 1\2	27\12	28\12	29\8
**Surgical procedure (*****n*****)**			
Circumcision or revision	12	16	10
Chordee or torsion repair	5	4	5
Hypospadias repair non-stented	6	5	9
Hypospadias repair stented	16	12	16
Surgical time (min)	41 ± 18	45 ± 21	45 ± 21

Pain scores in the PACU were statistically (*p* < 0.001) lower in the DPNB and caudal block groups compared to the fentanyl group during the first 20 min postoperatively, as detected by the FLACC score and for the first 30 min by the NRS score (Table 2). Pain scores were the lowest in the caudal group during this time period. After the first 30 min, pain scores were not different among the three groups for the rest of their PACU stay and throughout their hospital stay.

**Table 2:** PACU data.

**Method of analgesia**	**Caudal block** **(*n* = 38)**	**Dorsal penile nerve block** **(*n* = 32)**	**Fentanyl** **(*n* = 40)**	***p*-value among the groups**
Patients requiring MO in PACU (*n*)	5	14	32	
Time to1st MO administration (min)	18 ± 18	27 ± 22	23 ± 32	0.971
Range (min)	(0–40)	(0–80)	(0–170)	
MO doses: 1\2\3	5\0\0	10\4\0	16\12\4	<0.001
Total MO in PACU (mg/kg)	0.13 ± 0.35	0.55 ± 0.86	0.94 ± 0.83	<0.001
Time to fully awake (min)	54 ± 33	40 ± 39	36 ± 42	0.13
Range (min)	(0–120)	(0–150)	(0–140)	
O_2_ saturation at arrival to PACU (%)	97 ± 2	97 ± 4	96 ± 4	0.63
Range (%)	(92–100)	(80–100)	(80–99)	
O_2_ saturation at discharge (%)	98 ± 1	98 ± 2	98 ± 2	0.54
Range (%)	(95–100)	(93–100)	(95–100)	
Nausea/Vomiting (*n*)	2	0	5	0.09
**Pain and sedation scores in minutes after arrival to PACU (min)**				
FLACC 10	0.8 ± 2.1	1.5 ± 2.6	4.5 ± 4.2	<0.001
FLACC 20	0.4 ± 1.3	3.4 ± 3.5	2.8 ± 4.0	<0.005
FLACC 30	0.7 ± 1.5	1.7 ± 2.7	1.4 ± 2.7	0.16
FLACC 60	0.8 ± 1.6	0.5 ± 1.7	1.1 ± 2.3	0.49
NRS 10	0.8 ± 1.9	2.2 ± 3.6	3.7 ± 3.6	<0.001
NRS 20	0.6 ± 1.3	2.4 ± 3.5	2.7 ± 3.3	<0.005
NRS 30	0.6 ± 1.4	2 ± 3	1.2 ± 1.9	0.04
NRS 60	0.8 ± 1.2	1.8 ± 1.1	1.7 ± 2.4	0.32
SED 10	2.6 ±0.9	1.9 ± 1.2	1.3 ± 1.3	0.006
SED 20	2.3 ± 0.9	1.9 ± 1.2	1.3 ± 1.3	<0.005
SED 30	2 ± 1.2	1.3 ± 1.3	1.6 ± 1.3	0.05
SED 60	1.9 ± 5.6	0.9 ± 1.2	1.3 ± 1.4	0.48

*MO, morphine; PACU, post anesthesia care unit; FLACC, (Faces, Legs, Activity, Crying, Consolability) objective pain score from 0 - behavior signifies no pain to 10 - behavior signifies worst pain; NRS, numerical rating scale, subjective pain scale by parents from 0, no pain to 10 worst pain imaginable; SED, sedation score from 0-fully awake to three too sleepy to respond to touch*.

The relative risk for patients requiring morphine in the PACU as well as the total morphine consumed (mg) was lowest in the caudal group (*p* < 0.001) (Table 2). The time elapsed in the PACU to first morphine dose administration among those who required additional analgesia was not different among all groups (Table 2).

Sedation scores were significantly (*p* < 0.05) higher in the caudal group (Group 1) for the first 30 min in the PACU compared to the fentanyl or the DPNB group (Table 2); this measure paralleled the lower pain scores in the caudal group. There was no difference among the fentanyl and DPNB groups in sedation scores. Past 30 min into the PACU time of stay, the abovementioned higher caudal sedation score disappeared for the rest of the duration of the PACU course.

Nausea and vomiting in the PACU was not different between the study groups (*p* = 0.09). Respiratory rates as well as oxygen saturation (while breathing room air), both on arrival and on discharge from the PACU, were not different among the three groups. There was no statistical difference in time to eat, void or return to normal activity among the groups (Table 3).

**Table 3:** Ward data.

**Method of analgesia**	**Caudal** **(*n* = 38)**	**Dorsal penile nerve block** **(*n* = 32)**	**Fentanyl** **(*n* = 40)**	***p*-value**
Number of patients requiring Advil/Tylenol: (*n*)	14	13	19	0.67
Time to Advil/Tylenol: (min)	245 ± 89	205 ± 129	261 ± 175	0.6
Nausea/Vomiting (*n*)	3	3	8	0.27
Time to return to normal activity (min)	621 ± 625	526 ± 545	892 ± 945	0.11
Range	(60–1440)	(24–1440)	(60–2880)	
Time to return to normal eating (min)	133 ± 82	133 ± 73	134 ± 71	1
Range	(7–360)	(40–420)	(10–420)	
Time to return to normal voiding (min)	264 ± 295	195 ± 85	317 ± 270	0.25
Range	(60–1440)	(80–360)	(60–1440)	
FLACC 1 h post arrival to ward	0.7 ± 2.1	0.2 ± 1	0.6 ± 1.7	0.08
FLACC 4 h post arrival to ward	0.7 ± 2	3.6 ± 3.5	0.9 ± 2	0.06
NRS 1 h post arrival to ward	0.6 ± 1.2	0.5 ± 1.1	0.5 ± 1	0.2
NRS 4 h post arrival to ward	0.4 ± 1	0.6 ± 1.1	0.3 ± 0.9	0.01
SED 1 h post arrival to ward	0.6 ± 1.2	0.5 ± 1.1	0.5 ± 1	0.5
SED 4 h post arrival to ward	0.4 ± 1	0.6 ± 1.1	0.3 ± 0.9	0.7

*FLACC, (Faces, Legs, Activity, Crying, Consolability) objective pain score from 0-behavior signifies no pain to 10 - behavior signifies worst pain; NRS, numerical rating scale subjective pain scale by parents from 0 = no pain to 10 worst pain imaginable; SED, sedation score from 0-fully awake to three too sleepy to respond to touch*.

The telephone interview on POD 4 made with the designated parent, showed no differences in the patients' recovery: the time to normal eating, and the home activity patterns were not different in all groups. The number of children who required pain medications at home and their amounts were not different as well, as was the overall parental satisfaction scores (Table 4).

**Table 4:** Home data via interview on POD 4.

**Method of analgesia**	**Caudal block** **(*n* = 26)**	**Dorsal penile nerve block** **(*n* = 17)**	**Fentanyl** **(*n* = 25)**	***p*-value between fentanyl and blocks**
Total analgesics (doses)	2 ± 1.8	1.8 ± 2.6	3 ± 3.1	0.3
Overall satisfaction score	9.3 ± 1.6	9.5 ± 1	9.6 ± 0.6	0.5

*Satisfaction score subjectively scored by parents on a scale from 0 to 10, where 0 = least satisfied and 10 = most satisfied*.

## Discussion

This was a single center, prospective, randomized and double-blinded trial comparing three anesthetic techniques for penile surgery in children: general anesthesia with fentanyl or combined with either caudal or dorsal penile nerve block. We aimed at identifying the technique which would best control post-operative pain and allow optimal return to normal function over the first 4 days after pediatric penile surgery.

We found that during the first 30 postoperative min, both the caudal and penile blocks were associated with lower pain scores and narcotic requirements than the fentanyl group. Recovery of spontaneous voiding was earliest in the penile block group; however, this did not reach statistical significance. There was no difference in parental satisfaction scores or in the child's return to normal activity over four post-operative days.

Many studies have compared different options for pain management after pediatric penile surgery; however, to our knowledge, this is the first that also investigated differences in parental satisfaction during four post-operative days. The Cochrane Database Systematic Review in 2008 concluded that differences in analgesic need could not be detected between caudal, parenteral and penile block methods for circumcision, but that there is a need for more trials (1). More recently, Baird reviewed analgesic methods for pediatric hernia repair; there too, similar to our findings, they found no difference in analgesic outcome between caudal and peripheral blocks by 1 h after surgery (6). Contrary to our finding, Panda showed more effective analgesia up to 8 h after penile surgery with a DPNB and earlier recovery of normal eating compared to general anesthesia with fentanyl (7). A retrospective analysis of caudal vs. penile blocks with and without ultrasound guidance for circumcisions found that fewest caudal patients required intra-operative narcotics and that they had the longest time before requesting supplemental analgesia; the ultrasound guided block was as effective as the caudal in the PACU, but the landmark penile blocks required significantly more narcotics peri-operatively. Similar to our study the differences were recorded through the PACU period only (8).

Caudal blocks are the most common pediatric regional anesthesia used because it is easily performed, reliable and safe (4). There is a perceived preference to caudal blocks in younger pediatric patients in retrospective reports, as the motor block is less of a hindrance to their recovery (8). We had only one failed caudal block and found no difference in age distribution in this randomized and prospective study. We also found that motor block associated with the caudal blocks did not impact on recovery parameters. The likelihood that caudal block is associated with surgical complications in hypospadias surgery, as suggested by Saavedra-Belaunde (9), has been refuted by different studies (10) and meta-analysis (11). Rarely, when DPNB is used, the analgesics may infiltrate the tissues of the dorsal aspect of the penis and distort anatomical planes, which may cause technical difficulties and theoretically affect the procedure's success and final penile appearance. These aspects of caudal block and DPNB were not evaluated in this prospective study which tracked recovery parameters for the first 4 post-operative days.

We defined block failure as the need for supplemental IV fentanyl because of patient's response to surgery. Failure rate was higher in the DPNB group (13.5%) than in the caudal group (2.5%). We performed the DPNB using landmark technique rather than an ultrasound guided technique. Nevertheless, a more recent prospective study by Teunken comparing analgesic outcome using DPNB for circumcision via either the landmark or ultrasound technique found no difference in pain scores or in needs for analgesic supplementations intraoperatively or 24 h postoperatively (12). The failure rate in their study was somewhat higher than ours, 26% in the landmark group; and 36% in the ultrasound-guided group. Sandeman retrospectively compared landmark and ultrasound guided DPNB to caudal blocks in 216 pediatric circumcisions and found that 63% of patients with landmark placed DPNBs required intraoperative narcotics, compared to 5.5% of the caudal-based anesthetics and just 1.7% of the ultrasound placed DPNB. He also found that the landmark placed penile block patients required more morphine in the PACU compared to those with either caudal of ultrasound guided penile blocks (8). Chan and coauthors' retrospective study of more than 700 pediatric penile surgeries reported, similar to our study, that 18% of children treated with dorsal penile blocks required intraoperative supplemental narcotics as compared to 2% of caudal block children and that they had nearly 2.7 times the odds to have a pain score >3/10 in the PACU compared to those with caudal blocks (5).

Our finding that patients with penile blocks had a trend toward earlier return of spontaneous micturition is supported by Metzelder et al. who found less impairment of micturition after hypospadias repair with a DPNB compared to a caudal block (13). Wang et al. as well-reported that an ultrasound-guided dorsal penile nerve block provided comparable analgesia to the caudal block for circumcisions and was associated with a shorter time to spontaneous voiding (14).

This study bears with it several limitations. These include the small number of patients, which is the probable cause for the near statistically significant results. Children from a broad age range were included in this study. It is plausible that blocks may have different effectiveness in different age groups, such as pre-pubertal vs. post-pubertal boys. The small number of participants is not sufficient for sub-analysis of age groups. In addition, the telephone interview (POD 4) was accomplished in approximately half of the cases because many parents did not answer the calls. Their eventual interview upon their postoperative visit (7–10 days after surgery) was discarded since real-time data could not be analyzed with delayed responses. Some of the studied children required urethral stents, while others did not; however, they were equally distributed among the groups, so that results appear not to be biased toward one subgroup. Finally, there were some children who, despite an apparently adequate block during surgery, came to the PACU and required analgesia within the first 0–30 min. Although all were premedicated with midazolam, some of these children might have been agitated or crying on arrival to the PACU because of emergence from the inhaled anesthetics. Due to their high FLACC and parental VAS score, they were therefore medicated with morphine. Analgesia is part of the initial treatment of emergence agitation in non-communicative children (15) and might have introduced an error when analyzing the results.

In conclusion, post-operative pain was initially better controlled with regional anesthesia; however, once immediate analgesics were administered (within the first 30 min after arrival to the PACU), pain scores, analgesia requirements and 4-day recovery, as expressed by time to start eating and the return to normal activity, as determined by parents, were not different among all penile surgery groups of children.

## Data Availability Statement

The raw data supporting the conclusions of this article will be made available by the authors, without undue reservation.

## Ethics Statement

The studies involving human participants were reviewed and approved by Tel-Aviv Medical Center ethics committee. Written informed consent to participate in this study was provided by the participants' legal guardian/next of kin.

## Author Contributions

ME concept, design, analysis of data, and drafting of manuscript. YB-Y concept, design, interpretation of data, and drafting of manuscript. YK acquisition and analysis of data. RS interpretation and analysis of data. EA interpretation and analysis of data. JB-C concept, interpretation of data, and revision of manuscript. AW design, interpretation of data, and revision of manuscript. All authors contributed to the article and approved the submitted version.

## Conflict of Interest

The authors declare that the research was conducted in the absence of any commercial or financial relationships that could be construed as a potential conflict of interest.
